# Retraction: A deep learning-based ensemble for autism spectrum disorder diagnosis using facial images

**DOI:** 10.1371/journal.pone.0340328

**Published:** 2025-12-26

**Authors:** The PLOS One Editors

Following the publication of this article [1] concerns were raised with the dataset used in this study. Specifically, the dataset reportedly presents photographs of children with and without autism spectrum disorder. However, this diagnosis does not appear to have been validated. In addition, it is not clear whether the legal guardians of the children included in the dataset gave consent for these images to be distributed in a public dataset or used in academic research.

In light of the concerns pertaining to the ethics and the validity of the dataset underlying the study, the *PLOS One* Editors retract this article [1]. PLOS regrets that these issues were not identified prior to publication.

This article [1] was republished on December 16, 2025, to remove images of children’s faces in light of the concerns that have been raised.

This article [1] was removed from the *PLOS One* website on December 23, 2025. The article’s Copyright and Data Availability statements were updated at the time of retraction and removal, and the removed contents are no longer offered under the Creative Commons Attribution License.

TF, SA, MR, and MAI did not agree with the retraction. AJ and SMB either did not respond directly or could not be reached.
